# Healthcare-seeking behaviors for common childhood illnesses and associated factors among mothers/caretakers of under-five children in Haro Limu district, East Wollega, Oromia, Ethiopia: a cross-sectional study

**DOI:** 10.3389/fpubh.2025.1519027

**Published:** 2025-07-16

**Authors:** Shibru Fekadu Oljira, Emiru Merdassa, Zelalem Desalegn, Markos Desalegn

**Affiliations:** ^1^Nekemte Town Health Office, Oromia Regional Health Bureau, Nekemte, Ethiopia; ^2^Department of Public Health, Institute of Health Sciences, Wollega University, Nekemte, Oromia, Ethiopia

**Keywords:** common childhood illness, health seeking behavior, under five children, Haro Limu, East Wollega

## Abstract

**Background:**

Healthcare has gained increasing significance in the quest to achieve a healthy society. Timely healthcare-seeking can help reduce child mortality rates. In many developing countries, the health of children is closely tied to the healthcare-seeking behavior of their mothers/caretakers.

**Objective:**

This study aimed to assess the prevalence of healthcare-seeking behavior among mothers/caretakers for common childhood illnesses and to identify the associated factors in Haro Limu, East Wollega, Western Ethiopia, in 2019.

**Methods:**

A community-based cross-sectional study was conducted from 20 July to 30 September 2019 in the Haro Limu district. The required sample size was 459 mothers/caretakers of children under 5 years of age who had experienced a common childhood illness within the 4 weeks preceding the study. A multistage random sampling technique was used to select the study participants. Data were collected by health extension workers (HEWs) through face-to-face interviews using a structured questionnaire. The collected data were entered into EpiData (version 3.02) and exported to Statistical Package for the Social Sciences (SPSS) (version 20) for analysis. Binary logistic regression analysis was performed to identify the factors associated with healthcare-seeking behavior. The findings are presented using tables, pie charts, bar graphs, and narrative text.

**Results:**

A total of 459 mothers/caregivers were included in this study, achieving a 100% response rate. Among children under 5 years who had experienced a common childhood illness within the 4 weeks preceding the survey, 68% (95% CI:65–71.1%) sought care from health facilities. Perceived severity of illness (AOR = 4.23, 95% CI: 2.27, 7.88), husband’s education level of secondary or higher (AOR = 2.70, 95% CI: 1.29, 5.64), and belonging to the richest household category (AOR = 2.40, 95% CI: 1.06, 5.44) were identified as independent predictors of the healthcare-seeking behavior of mothers/caretakers.

**Conclusion:**

A considerable proportion of respondents did not seek care for their sick children at health facilities. It is essential to educate families and raise awareness in the community regarding the importance of seeking healthcare.

## Introduction

Healthcare has gained greater significance today in the effort to achieve a healthy society. Health affects all aspects of human life and has broader implications for an individual’s social life. Therefore, improving the health status of people is challenging not only for medical practitioners but also for social scientists (1). Achieving universal health coverage means ensuring that everyone can access the essential health services they need (2). All children deserve high-quality medical care when they are ill (3). However, worldwide, a significant number of under-five children die without receiving any healthcare (4). The concept of studying healthcare-seeking behaviors has evolved over time. Today, it has become a tool for understanding how people engage with healthcare systems within their respective socio-cultural, economic, and demographic contexts (1).

Healthcare-seeking behavior is any action undertaken by individuals experiencing health issues to seek appropriate medical treatment; it is a specific aspect of help-seeking behavior (5). People differ in their willingness to seek help from healthcare services (1). Healthcare-seeking behavior is preceded by a decision-making process that is influenced by individual and/or household behavior, community norms, and expectations, as well as provider-related characteristics and behavior (5). Many factors such as sex, age, type of illness, access to services, and perceived quality of care influence healthcare-seeking behavior (1).

Common childhood illnesses refer to diseases that significantly contribute to child morbidity, disability, and mortality around the world. An estimated 7 million children under the age of 15 years died in 2022, of which 4.9 million were children under the age of five (3). However, a large number of children die without receiving appropriate treatment (4). The most common childhood illnesses leading to death are complications during birth, pneumonia, diarrhea, neonatal sepsis, and malaria, which can be prevented (6–8). Globally, pneumonia and diarrhea accounted for a combined 23% of under-five mortality, resulting in approximately 1.17 million deaths in 2021 (4). Over 70% of these deaths were concentrated in 15 countries, including Ethiopia (5). These deaths are highly concentrated in the poorest regions and countries. Nearly 90% of deaths due to pneumonia and diarrhea occur in sub-Saharan Africa and South Asia (6).

Timely and appropriate care sought by caregivers could reduce child mortality by 20% (7). To save children, it is essential for parents to prioritize seeking adequate medical attention (8). This study aimed to identify healthcare-seeking behavior and its associated factors among women who have under-five children.

## Materials and methods

A household-based cross-sectional study was conducted in the Haro Limu district, which is one of the 17 districts in East Wollega, Oromia, Western Ethiopia. The study was conducted from April 2019 to March 2020.

The source population consisted of all mothers/caregivers living in the Haro Limu district who had under-five children with common childhood illnesses within the 4 weeks preceding the survey. The study population included all mothers/caregivers of under-five children with common childhood illnesses within the 4 weeks before the survey residing in the selected kebeles. A multistage random sampling technique was used. The district is classified into urban and rural areas. One of the two urban kebeles and nine of the 15 rural kebeles were randomly selected. The sample size was determined proportionally based on the number of households in each kebele. Eligible mothers/caretakers were selected using systematic random sampling, and every other household was considered. If multiple eligible mothers/caregivers (those with children under five) resided in a household, one was randomly selected as the study participant.

All mothers/caretakers who had under-five children with a history of common childhood illness within the 4 weeks before the survey, residing for more than 6 months in households within the selected kebeles were included in the study. Mothers/caregivers with serious mental health problems and those who were very sick during the survey were excluded from the study because it was believed that they would be unable to provide the necessary information.

The sample size was determined using a single population proportion formula. The following assumptions were considered: a margin of error of 5%, a 95% confidence level, and a design effect of 1.5. Based on previous data, the percentage of mothers who sought care from health institutions for common childhood illnesses was 74.6% (9). The total sample size calculated was 459.

A structured questionnaire was developed to assess the sociodemographic characteristics of the mothers/caretakers, history of childhood illnesses, mothers/caretaker’s healthcare-seeking behavior for common childhood illnesses, and information about health facilities. The questionnaire was adapted from various reviewed literature sources (10–14). It was initially prepared in English, then translated into Afan Oromo and back-translated into English by language professionals. The questionnaire was pretested among 23 mothers in the Tutine kebele of the Haro Limu district, which has a similar sociodemographic background with the selected kebele. Data were collected by 10 trained health extension workers (HEWs), and 2 supervisors (BSc. health workers) were selected and involved in the data collection process. Training was provided to the data collectors and supervisors for 2 days on how to complete the questionnaire. Both the data collectors and supervisors practiced using the questionnaire during the training.

Data were collected using face-to-face interviews. The collected data were checked for completeness and consistency on a daily basis by a supervisor. Any filled questionnaire with issues was corrected by revisiting the household. The data were entered into EpiData (version 3.02) for cleaning, then the data were exported to Statistical Package for the Social Sciences (SPSS) (version 20.0) for analysis. Descriptive statistics, frequencies, percentages, and means were used to describe the study variables. Independent variables with a *p*-value of <0.2 were included in the multivariable model to control for all possible confounders (15). The Hosmer–Lemeshow goodness-of-fit test was used to assess the model’s fitness. Adjusted odds ratios (AOR) with 95% confidence intervals (95%CIs) were estimated to measure the strength of the association between dependent and independent variables and to identify factors associated with healthcare-seeking behavior among the mothers/caregivers for common childhood illnesses. The results of the multivariable logistic regression models were interpreted using AOR with 95% confidence intervals (95%CI), and variables with a *p*-value less than 0.05 were considered statistically significant.

Ethical clearance was obtained from the Institutional Review Board of the Institute of Health Science Committee (IRBIHSC) at Wollega University. Additionally, support letters were obtained from the East Wollega Health Office to the Haro Limu District Health Office, and subsequently from the Haro Limu District Health Office to the selected kebeles. Informed, voluntary written consent was obtained from each participant. Confidentiality and the rights of the respondents were respected, and each participant was informed about the purpose of the study. The respondents were assured that all information provided would remain confidential.

## Results

### Sociodemographic characteristics of respondents

A total of 459 mothers/caregivers were included in this study, yielding a 100% response rate. The median age of the interviewed respondents was 30 years (with interquartile range of 6), ranging from 20 to 54 years. Of the total participants, 422 (91.9%) were currently married. Regarding the respondents’ religion, 313 (68.2%) were Protestants, followed by Muslims, who accounted for 71 participants (15.5%). More than half of the study participants, 267 (58.2%), had no formal education, while 140 participants (30.5%) had completed their primary education. Nearly half of the mothers/caregivers, 221 (48.1%), were farmers, followed by 177 participants (38.6%) who were unemployed. Among the participants interviewed, 282 (61.4%) lived in households with more than five family members (Table 1).

**Table 1:** Socio-demographic characteristics of respondents (*n* = 459) in Haro Limu district, Oromia regional state, Western Ethiopia, 2019.

Variables	Category	Frequency	Percent
Residence	Urban	51	11.1
	Rural	408	88.9
Age of mothers in year	<25	89	19.4
	25–34	280	61.0
	≥35	90	19.6
Marital status of mother	Married	422	91.9
	Not married	37	8.1
Religion	Protestant	313	68.2
	Orthodox	62	13.5
	Muslims	71	15.5
	Wakefata	13	2.8
Educational status of mother	No formal education	267	58.2
	Primary school	143	31.2
	Secondary and above	49	10.6
Educational status of husband	No formal education	189	45
	Primary school	147	35
	Secondary school and above	84	20
Family size in the house hold	≤ 5	101	22
	>5	358	78
Wealth Index	Richest	92	20.04
	Rich	92	20.04
	Middle	92	20.04
	Poor	91	19.8
	Poorest	92	20.04
Number of <5 children per mother	One child	224	48.8
	Two and above	235	51.2

### Child health-related characteristics and healthcare-seeking practices of mothers/caregivers

**Figure 1:**
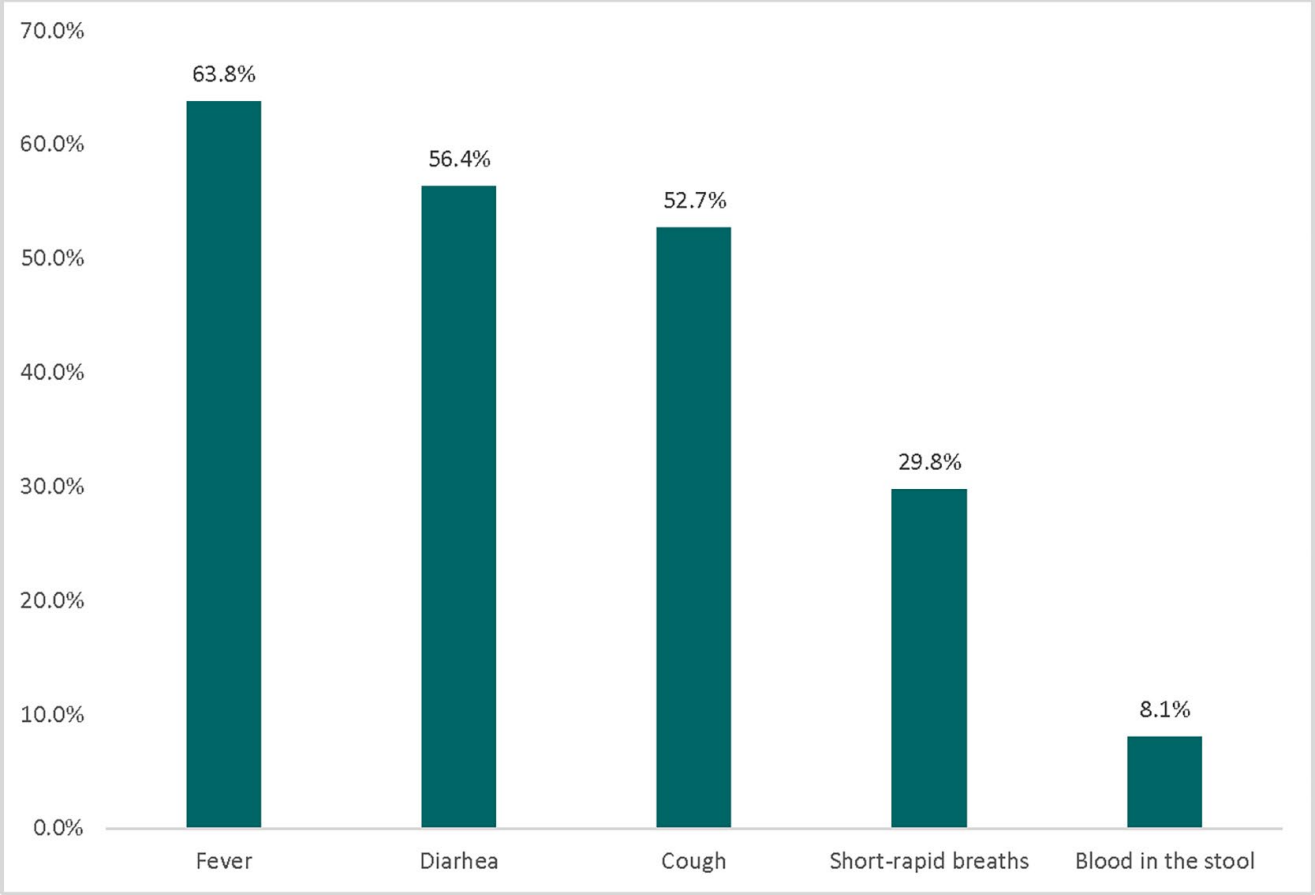
The percentage distribution of childhood illness reported by mothers/caretakers, Haru Limu, East Wollega, in 2019.

Of the total 459 sick children, 392 (85.4%) were in the 12–59 months age group. Nearly half, 231 (50.3%), of the children had two or more disease symptoms. Among the 459 sick children, 242 (52.7%) had cough symptoms, 259 (56.4%) had diarrhea, and 293 (63.8%) had fever (Figure 1). Regarding healthcare place preference, 397 (86.5%) of the participants preferred to seek healthcare from a health institution when their child experienced illness.

**Figure 2:**
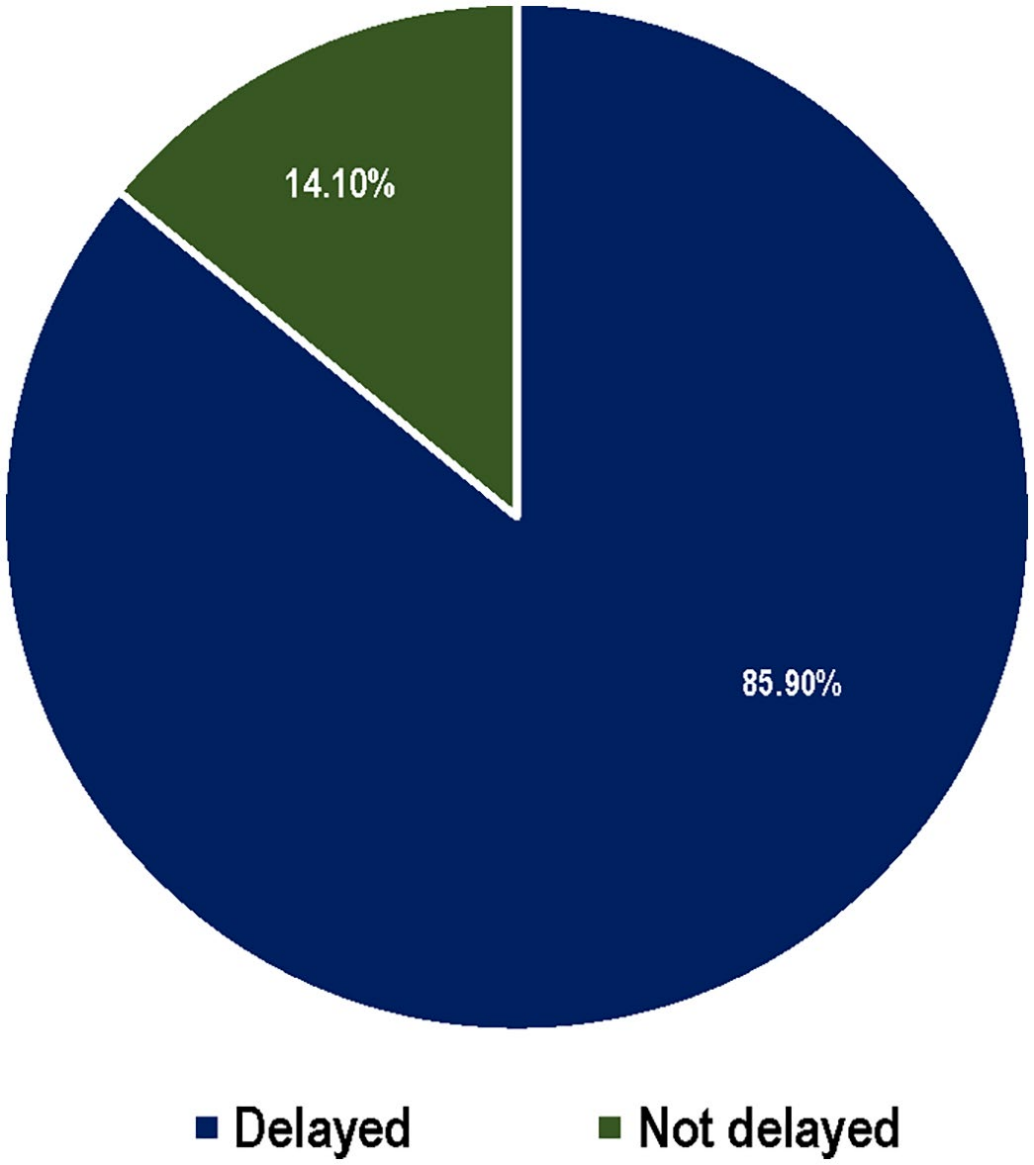
The percentage distribution of mothers/caretakers’ care seeking behavior of with children under five old who had experienced common childhood illness within the four weeks preceding, Haro Limu, Haro, East Wollega, in 2019.

The majority of the respondents, 268 (85.9%), sought delayed healthcare for their sick children (Figure 2). A large proportion of children who experienced illness sought care from health facilities. Regarding the perceived cost of treatment at health facilities, the majority of the respondents, 308 (67.1%), reported that it was not expensive. A total of 147 mothers did not seek healthcare from health facilities. The reasons for not seeking healthcare were as follows: 65 (44.2%) of the respondents perceived the illness as not serious and 27 (18.4%) cited a lack of money (Table 2).

**Table 2:** Child health related characteristics and health seeking practice of mothers/caregivers (*n* = 459) in Haro Limu district, Oromia regional state, Western Ethiopia, 2019.

Variables	Category	Frequency	Percent
Age of child in month	0–11	67	14.6
	12–59	392	85.4
Sex of child	Male	225	49
	Female	234	51
Number of symptoms the child illness	One	228	49.7
	Two and more	231	50.3
Perceived severity of illness	Yes	341	74.3
	No	118	25.7
Mothers place of preference to seek care	Health institution	397	86.5
	Others (home remedy, traditional healer)	62	13.5
Perceived cost of child treatment	Not expensive	308	67.1
	Expensive	151	32.9
Reason, for not visiting health facility	Illness was not serious	65	44.2
	Lack of money	27	18.4
	Absence of health facility in the area	17	11.6
	Didn’t have time	9	6.1
	Others	29	19.7

### Predictors of healthcare-seeking behavior among mothers

To identify the predictors of healthcare-seeking behavior among the mothers, a multivariate binary logistic regression analysis was performed. The following three variables: husbands’ educational status, household wealth index, and perceived severity of the disease were significantly associated with the mothers/caregivers’ healthcare-seeking behavior.

The results of the multivariable logistic regression analysis showed that the mothers/caregivers whose husbands had completed secondary education or higher were 2.70 times more likely to seek healthcare than those whose husbands were illiterate (AOR=2.70, 95%CI: 1.29, 5.64). The participants from the richest households were 2.40 times more likely to seek healthcare than those from the poorest households (AOR=2.40, 95%CI: 1.06, 5.44). In addition, the participants who perceived their child’s illness as severe were 4.23 times more likely to seek healthcare than the participants who perceived the illness as not severe (AOR=4.23, 95%CI: 2.27, 7.88) (Table 3).

**Table 3:** Multivariate analysis of health care seeking behavior for common childhood illnesses (*n* = 459) in Haro Limu district, East Wollega zone, Oromia regional state, 2019.

Variable	Category	COR	AOR
Residence	Urban	3.96 (1.65, 9.51)	1.43 (0.42, 4.88)
	Rural	1	
Husband’s education level	No formal education	1	
	Primary school	1.02 (0.65, 1.60)	0.95 (0.59, 1.54)
	Secondary and above	3.15 (1.59, 6.21)	2.70 (1.29, 5.64) ^**^
Mother/caretaker’s occupation	Government employee	0.99 (0.33, 2.99)	0.26 (0.06, 1.13)
	Merchant	2.09 (0.91, 4.77)	0.96 (0.34, 2.76)
	Farmer	0.81 (0.53, 1.23)	1.03 (0.61, 1.72)
	Unemployed	1	
Sex of the child	Male	0.70 (0.47, 1.04)	0.64 (0.41, 1.02)
	Female	1	
Number of symptoms	One	1	
	Two and above	1.76 (1.19, 2.62)	1.08 (0.61, 1.90)
Wealth index	Richest	4.48 (2.22, 9.04)	2.40 (1.06, 5.44) ^*^
	Rich	1.66 (0.91, 3.03)	1.08 (0.54, 2.19)
	Middle	1.66 (0.91, 3.03)	1.21 (0.61, 2.41)
	Poorer	1.48 (0.82, 2.69)	1.15 (0.58, 2.27)
	Poorest	1	
Perceived severity	Yes	3.13 (1.83, 5.36)	4.23 (2.27, 7.88) ^***^
	No	1	
Perceived cost of Treatment	Expensive	1	
	Not expensive	1.91 (1.01, 3.66)	2.00 (0.98, 4.09)

^*^ Statistically significant at *p* < 0.05. ^**^ statistically significant at *p* < 0.01. ^***^ statistically significant at *p* < 0.001.

## Discussion

In this study, 68% of the mothers sought healthcare for common childhood illnesses (95% CI: 64.95, 71.05%). This proportion is comparable to findings from the Lume district in East Shewa, Oromia (69.9%), and Bahirdar (72.7%), where sick children also sought care from health facilities (14, 16). However, this study’s rate is higher than those reported in Northern Ethiopia and Yemen, where only 48.8 and 51.42% of mothers with children under 5 years of age sought healthcare, respectively (17, 18). Conversely, the rate observed in this study is lower than those reported from the Jeldu district and Bure district, Northwest Ethiopia, where 74.6 and 79.3% of mothers sought care (9, 19). These differences may be related to socio-demographic factors such as husbands’ education level, household wealth, study duration, and healthcare availability.

Delays in seeking healthcare were evident, with only 14.1% of the mothers seeking care within 24 h of recognizing their child’s illness. This finding aligns with studies conducted in the Jeldu and Lume districts (9, 20), as well as a study in Yemen (18), all of which showed healthcare-seeking delays. The main reasons for not seeking care were the perception that the illness was not severe (44.2%) and financial constraints (18.4%). This finding aligns with research conducted in Dangila town (21) but contrasts with studies conducted in the Lume and Jeldu districts, where financial issues were the primary reason for inaction.

The multivariate logistic regression analysis indicated that the husband’s educational status influenced the mothers’ healthcare-seeking behavior. The mothers whose husbands had attained secondary education or higher were more likely to seek healthcare than those with illiterate husbands. This finding aligns with studies conducted in Bure, Northwest Ethiopia, but differs from findings in Jeldu and Lume (9, 20), where no such association was found, likely due to cultural beliefs.

The study demonstrated that the mothers who perceived their child’s illness as severe were more likely to seek healthcare than those who did not. This finding is consistent with studies conducted in the Jeldu and Lume districts. In addition, the mothers from wealthier households were more inclined to seek care, a finding supported by research in the Bure district and rural Tanzania (19, 22). The study also found that the mothers/caregivers from the richest households were more likely to seek healthcare than those from the poorest households. The finding is consistent with studies conducted in the Bure district and rural Tanzania, where participants from the richest households sought care more frequently compared to the poorest participants (19, 22). Some limitations should be considered when interpreting these findings. First, important variables such as the husband’s awareness of symptoms and signs in under-five children were not included. Second, this study used a cross-sectional design, in which causal associations cannot established.

## Conclusion and recommendation

This study showed that the education level of husbands, household wealth index, and perceived severity of illness are independent predictors of mothers’ healthcare-seeking behavior for common childhood illnesses. To improve healthcare-seeking behavior among mothers/caregivers, health promotion activities should focus on enhancing mothers’ and caregivers’ perceptions of illness severity and raising husbands’ awareness through targeted education and awareness campaigns.

## Data Availability

The raw data supporting the conclusions of this article will be made available by the authors, without undue reservation.
